# Takotsubo Syndrome in the Setting of Pacemaker Implantation: Results From a Multicenter National Prospective Registry

**DOI:** 10.31083/RCM39440

**Published:** 2025-09-28

**Authors:** Gonzalo García-Martí, Ravi Vazirani, Óscar Vedia, Agustín Martín-García, Aitor Uribarri, Miguel Corbí-Pascual, Emilia Blanco-Ponce, Juan M. Escudier Villa, Rafael Sánchez-Del Hoyo, Clara Fernández Cordón, Manuel Almendro-Delia, Victor M Becerra, Alberto Pérez Castellanos, Marta Guillen Marzo, Beatriz Alonso, Fernando Alfonso, Iván J. Núñez-Gil

**Affiliations:** ^1^Cardiology Department, Hospital Clínico San Carlos, IdISSC, 28040 Madrid, Spain; ^2^Cardiology Department, Hospital Universitario de Torrejón, 28850 Madrid, Spain; ^3^Cardiology Department, Hospital Clínico Universitario de Salamanca, 37007 Salamanca, Spain; ^4^Cardiology Department, Hospital Universitario Vall d´Hebron, 08035 Barcelona, Spain; ^5^Cardiology Department, Hospital General Universitario de Albacete, 02006 Albacete, Spain; ^6^Cardiology Department, Hospital Universitario Arnau de Vilanova, 25198 Lérida, Spain; ^7^Cardiology Department, Hospital Universitario Puerta de Hierro, 28222 Madrid, Spain; ^8^Research Methodological Support Unit and Preventive Department, Hospital Clínico San Carlos, IdISSC, 28040 Madrid, Spain; ^9^Cardiology Department, Hospital Clínico Universitario de Valladolid, 47003 Valladolid, Spain; ^10^Cardiology Department, Hospital Universitario Virgen de Macarena, 41009 Sevilla, Spain; ^11^Cardiology Department, Hospital Regional Universitario de Málaga, 29010 Málaga, Spain; ^12^Cardiology Department, Hospital Universitario Son Espases, 07120 Palma de Mallorca, Spain; ^13^Cardiology Department, Hospital Universitario Joan XXIII, 43005 Tarragona, Spain; ^14^Cardiology Department, Hospital General Universitario Gregorio Marañón, 28007 Madrid, Spain; ^15^Cardiology Department, Hospital Universitario La Princesa, 28006 Madrid, Spain

**Keywords:** takotsubo syndrome, pacemaker implantation, prognosis, registry

## Abstract

**Background:**

There is evidence that pacemaker implantation can trigger Takotsubo syndrome (TTS). However, limited information is available on the prognosis of TTS caused by this trigger, so our study aims to elucidate the clinical features, presentation, and prognostic factors associated with this syndrome in this specific situation.

**Methods:**

We analyzed a group of patients with TTS triggered by pacemaker implantation (n = 41), including consecutive cases from the multicenter registry on takotsubo syndrome (RETAKO) and patients identified through a systematic literature search, and compared them to the general RETAKO cohort (n = 1559). We performed a 1:3 propensity score matching (PSM) based on dyslipidemia, diabetes mellitus, smoker/ex-smoker status, syncope, angina, vagal symptoms, and physical/mixed trigger, generating two balanced groups.

**Results:**

Compared to other triggers, TTS associated with pacemaker implantation was linked to a longer corrected QT interval (551.2 ms vs. 502.5 ms, *p* = 0.005), lower left ventricular ejection fraction (34.8% vs. 47.3%, *p* < 0.001), a higher proportion of acute kidney injury (29.3% vs. 11.0%, *p* = 0.001), and an increased rate of cardiogenic shock (20.6% vs. 8.8%, *p* = 0.029). However, there were no differences in all-cause mortality (12.2% vs. 13.1%, *p* = 0.858) or TTS recurrence (0.0% vs. 3.9%, *p* = 0.639). After PSM, the previously observed differences were no longer present, with no significant differences in death or recurrences.

**Conclusions:**

TTS following pacemaker implantation predominantly presents with greater rates of cardiogenic shock and acute kidney injury, without differences in all-cause mortality or TTS recurrence. After PSM, no differences were found regarding cardiovascular outcomes, suggesting that the physical nature of the trigger could account for the initial differences observed.

## 1. Introduction

Takotsubo syndrome (TTS), first described in Japan in the early 1990s [1], consists of a transient dysfunction of the left ventricle, more common in postmenopausal women.

Secondary forms of TTS (i.e., those associated with a physical or mixed trigger) have been described to have a worse prognosis than primary forms (i.e., those idiopathic or associated with a psychological stress) [2, 3]. Pacemaker implantation constitutes a physical stress situation for the patient. If the reason for pacemaker implantation is an acute advanced atrioventricular block, this process can be an even greater stressor, as it triggers low cardiac output [4].

The first case report of TTS after pacemaker implantation was published in 2006 [5]. Niewinski *et al*. [6] published a series of nine cases of TTS after pacemaker implantation in 2020. Two years later, Strangio *et al*. [7] conducted a systematic review of 28 published cases, with limited clinical information.

Our objective was to elucidate the clinical characteristics and cardiovascular outcomes of patients affected by TTS in the context of pacemaker implantation, using published cases from the literature and from the multicenter registry on takotsubo syndrome (RETAKO) to generate a cohort larger than those previously studied; and to compare them with the general RETAKO cohort, as well as with patients with physical or mixed trigger, using propensity score matching (PSM).

## 2. Material and Methods

We conducted this study in accordance with the Helsinki Declaration and approval was granted by the Ethics Committee of the coordinating center. All patients provided signed informed consent.

### 2.1 Registry Data

We utilized the national multicenter registry of TTS: RETAKO, supported by the Ischemic Heart Disease and Acute Cardiovascular Care Section of the Spanish Society of Cardiology, initiated on January 1st, 2012. It is an ambispective multicenter registry developed in 36 centers: retrospective (from 2002 to 2012) and prospective (from 2012 to the present). Its rationale and design have been previously described [8].

The inclusion criteria for this registry are the revised Mayo Clinic criteria which include study of coronary anatomy to rule out obstructive coronary disease or acute plaque rupture. These criteria also include regional wall motion abnormalities that do not correspond to a specific coronary territory with transient changes on the electrocardiogram or modest elevation of troponin. Once patients were selected, their data were collected, anonymized, and transferred to a central database.

Patient follow-up after discharge could be conducted either remotely or in-person, with both patients and their families. The outcomes were pre-specified and were adjudicated by two experienced local investigators (I.N., O.V.). The same two experienced researchers carefully evaluated each case to assess the temporal relationship between pacemaker implantation and the development of TTS. Our predefined primary outcomes were all-cause mortality and the recurrence of TTS. As secondary outcomes, we established: the degree of left ventricular dysfunction (using the left ventricular ejection fraction (LVEF)), the length of the corrected QT interval (cQT), cardiogenic shock, in-hospital readmissions, acute kidney injury and intraventricular thrombus formation.

Thus, 21 patients from the RETAKO registry who suffered from TTS during the placement of a pacemaker; either immediately after or in the following 72 hours (as described in the literature [5, 9]), were included in the TTS group within the context of pacemaker implantation. The remaining 1559 patients were included in the group of TTS due to any other causes and served as a reference group.

### 2.2 Review Methods

For our study, we conducted a systematic review in March 2024 of TTS in the context of pacemaker implantation using the PubMed database, including the following terms: “Takotsubo”, “broken heart syndrome”, “stress cardiomyopathy”, “stress-induced cardiomyopathy”, “transient left ventricular ballooning” and “apical ballooning”. We combined these terms with: “pacemaker” and “pacemaker implantation”. We only included patients over 18 years old who met the revised Mayo Clinic criteria for the diagnosis of TTS.

**Fig. 1.**
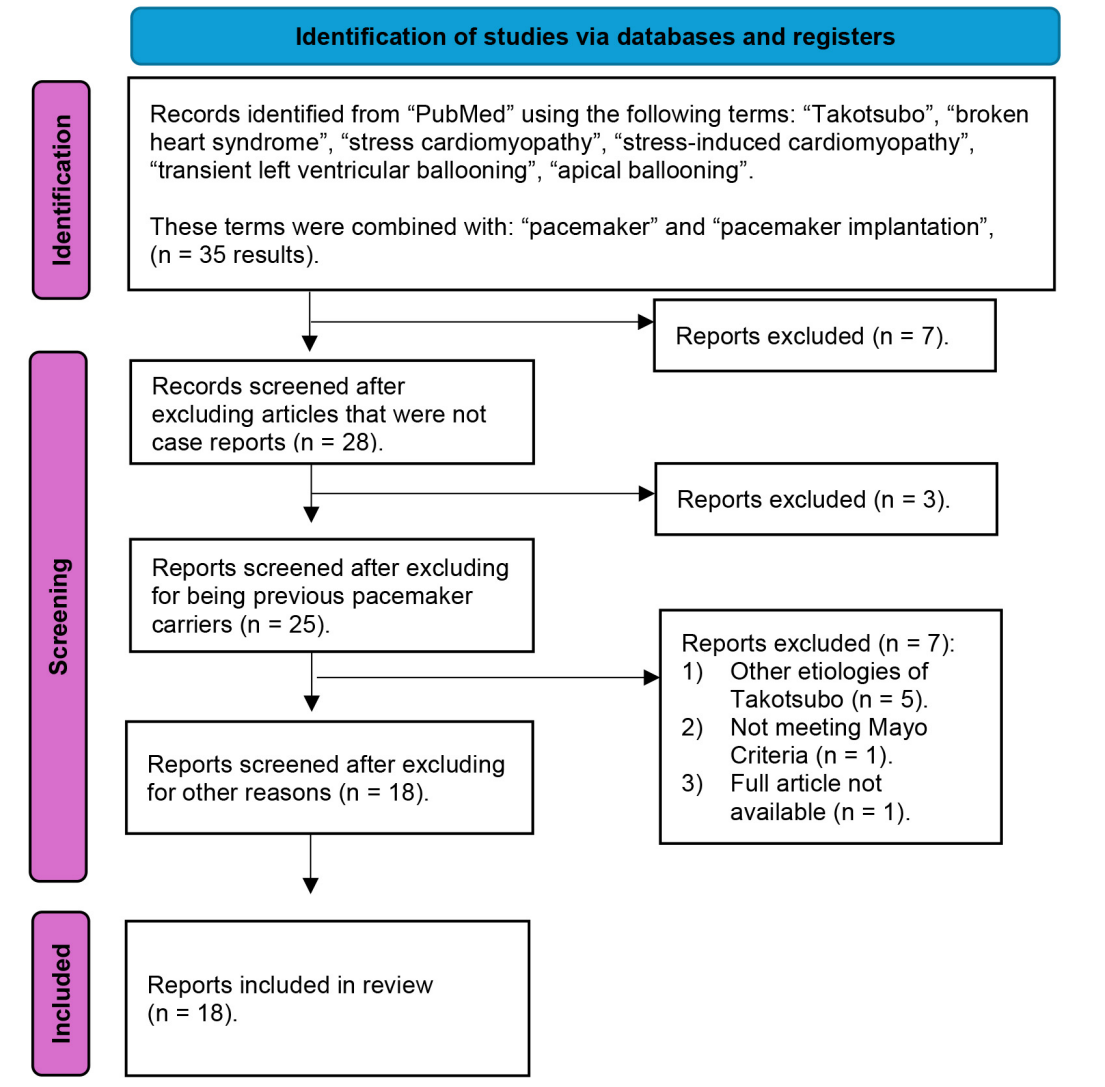
**PRISMA flowchart of the systematic review of the literature**. This figure shows the systematic literature review conducted in March 2024 in the PubMed database. A series of terms related to takotsubo syndrome (TTS) were combined with other terms related to pacemaker. Of the 35 results obtained, 17 were excluded. Finally, a total of 18 articles, including 20 patients, were selected for our study.

Fig. 1 summarizes the selection process and a detailed PRISMA flow diagram of the review. Finally, we selected 18 articles that met the established inclusion criteria, encompassing a total of 20 patients [5, 9, 10, 11, 12, 13, 14, 15, 16, 17, 18, 19, 20, 21, 22, 23, 24, 25].

Clinical information of the case reports from the patients used for this systematic review can be found in the **Supplementary Table 1**.

### 2.3 Group Formation

**Fig. 2.**
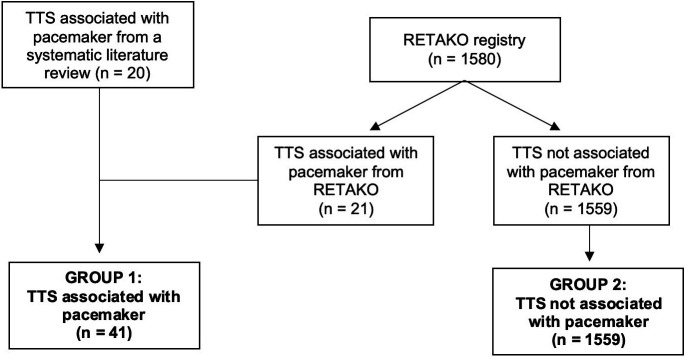
**Flowchart of the formation of the study groups**. The first group of 41 patients with Takotsubo syndrome (TTS) associated with pacemaker implantation was formed, including 20 patients identified through the systematic literature review and 21 from the national multicenter registry on takotsubo syndrome (RETAKO). The second group consisted of 1559 patients from RETAKO who presented with TTS due to a different cause.

Two groups were produced: (1) a group of 41 patients with TTS in the context of pacemaker implantation, consisting of 21 patients from the RETAKO registry and 20 patients from our systematic review; (2) a second group comprised of 1559 patients with TTS due to other triggers, all extracted from the RETAKO registry as described in Fig. 2.

### 2.4 Statistical Analysis

Continuous variables were expressed as either mean ± standard deviation or median (interquartile range), as appropriate, and categorical variables as percentages. Group comparisons were conducted using Pearson’s chi-square or Fisher’s exact test for categorical variables and Mann-Whitney for continuous variables. A 2-tailed *p* < 0.05 was considered statistically significant.

Statistical analysis and graphing were carried out using the Office 365 package (Microsoft, Redmond, WA, USA), SPSS software v.26 (IBM, Chicago, IL, USA) and R statistical software v. 4.3.1 (R Foundation for Statistical Computing, Vienna, Austria).

Additionally, we performed a 1:3 PSM using the previously mentioned groups based on the following variables: dyslipidemia, diabetes mellitus, smoker/ex-smoker status, syncope, angina, vagal symptoms, and physical or mixed trigger.

The adjusted incidence of clinical events during follow-up was assessed using the Kaplan-Meier method after PSM (calculated from hospital discharge to the last follow-up or death), with group comparisons made via the log-rank test.

Moreover, we conducted a sensitivity analysis between patients with TTS following pacemaker implantation from the RETAKO registry and those obtained from the literature (**Supplementary Table 2**), to assess for relevant differences between the two groups that could bias the data pooling.

As including the variables “syncope” and “angina” in the initial PSM model could have introduced confounding, we constructed a revised PSM that omits these two covariates while preserving all other baseline factors. The results of this analysis are presented in **Supplementary Table 3**. We also presented an analysis of the pacemaker indication, the type of pacemaker, and the timing of implantation in **Supplementary Table 4**.

## 3. Results

The baseline clinical characteristics and comorbidities are detailed in Table 1. The overall percentage of TTS in the context of pacemaker implantation within the RETAKO cohort was 1.3%. There were no differences in terms of age between patients with TTS associated with having pacemaker or not. Among patients with TTS, the proportion of females did not differ significantly between the two groups (78.0% vs. 86.7%, *p* = 0.112). Moreover, the burden of comorbidities was quite similar in both groups, although there was a higher percentage of patients with diabetes mellitus in the group associated with pacemaker implantation. No significant differences in cancer prevalence were observed between the groups.

**Table 1.** **Comparison of baseline clinical characteristics, comorbidities and cardiovascular outcomes between both groups**.

		TTS due to pacemaker implantation (n = 41)	TTS not due to pacemaker implantation (n = 1559)	*p*
	Age (years)	73.63 ± 7.82	70.52 ± 11.99	0.200
	Female	32/41 (78.0)	1345/1552 (86.7)	0.112
Comorbidities	Hypertension	25/34 (73.5)	1002/1491 (67.2)	0.437
	Dyslipidemia	11/35 (31.4)	694/1467 (47.3)	0.063
	Diabetes Mellitus	13/36 (36.1)	301/1474 (20.4)	0.022
	Smoker/Ex-smoker	8/35 (22.9)	399/1485 (26.9)	0.596
	Ischemic Heart Disease^1^	3/25 (12.0)	94/1412 (6.7)	0.236*
	Hepatopathy	1/22 (4.5)	56/1468 (3.8)	0.579*
	Cancer	2/41 (4.8)	99/1559 (6.4)	0.570*
TTS clinical presentation	Syncope	12/41 (29.3)	115/1466 (7.8)	<0.001
	Angina	12/41 (29.3)	936/1444 (64.8)	<0.001
	Vagal symptoms	12/40 (30.0)	641/1472 (43.5)	0.088
	Dyspnea	19/34 (55.9)	460/1110 (41.4)	0.093
	Palpitations	3/40 (7.5)	111/1462 (7.6)	1.000*
	Physical/mixed trigger	41/41 (100.0)	530/1159 (34.0)	<0.001*
	Apical akinesia^2^	34/41 (82.9)	1161/1558 (74.5)	0.221
Cardiovascular outcomes	LVEF (%)^3^	34.8 ± 8.5	47.3 ± 12.6	<0.001
	cQT interval (ms)^4^	551.2 ± 72.1	502.5 ± 70.1	0.004
	Acute kidney injury	12/41 (29.3)	159/1447 (11.0)	0.001
	Pulmonary edema	7/41 (17.1)	135/1454 (9.3)	0.102
	Cardiogenic shock	7/34 (20.6)	97/1104 (8.8)	0.029
	Intraventricular thrombus	2/41 (4.9)	41/1457 (2.8)	0.331*
	Readmission	1/35 (2.9)	137/1245 (11.0)	0.167*
	Follow-up (months)^5^	4.0 (1.3–19.0)	19.0 (5.0–52.0)	<0.001
	TTS recurrence	0/35 (0.0)	48/1245 (3.9)	0.639*
	In-hospital death	1/41 (2.4)	31/1559 (2.0)	0.568*
	All-cause mortality	5/41 (12.2)	205/1559 (13.1)	0.858

Categorical variables are reported as n/N (percentage). Continuous variables are presented as mean ± standard deviation or median (interquartile range), as appropriate. Pearson’s chi-square test was applied to categorical data, and the Mann-Whitney test to continuous data. Variables marked with an asterisk (*) were analysed using Fisher’s exact test. Abbreviations: TTS, takotsubo syndrome; LVEF, left ventricular ejection fraction; cQT, corrected QT interval. ^1^Regardless of its severity. ^2^Patients were classified based on whether it presented in the typical form of TTS (apical akinesia) or an atypical form (basal or mid-segment akinesia). ^3^Left ventricular ejection fraction (LVEF) is expressed as a percentage according to the biplane Simpson method by echocardiography. ^4^The corrected QT interval (cQT) is expressed in milliseconds using Bazett’s correction formula. ^5^Follow-up is expressed in months as median and interquartile range.

Regarding clinical presentation on hospital admission, syncope was more frequent in the group associated with pacemaker implantation (29.3% vs. 7.8%; *p* < 0.001). However, in the group not associated with pacemaker implantation, angina was present in a higher percentage of patients: 29.3% vs. 64.8% (*p* < 0.001). In the group of patients where TTS was not associated with pacemaker insertion, vagal symptoms were more frequent, whereas in the pacemaker group, dyspnea was more common; however, these differences were not statistically significant.

In both groups, there was a higher representation of the typical TTS pattern (apical akinesia with basal hypercontractility), with no significant differences (82.9% vs. 74.5%, *p* = 0.221). The representation of secondary forms of TTS (with a physical/mixed trigger) was 34.0% in the group not associated with pacemaker implantation.

Regarding the cardiovascular outcomes in the two groups: in the TTS associated with pacemaker implantation group, the LVEF was severely depressed (34.8%) and significantly lower (*p* < 0.001) than in the TTS group not associated with pacemaker implantation (47.3%). It was also observed that in the first group, the cQT interval was significantly longer than in the second group (551.2 ms vs. 502.5 ms, *p* = 0.005), although both groups were above the normal limits.

In the TTS associated with pacemaker group, a higher percentage of cardiogenic shock was observed, at up to 20.6% compared to 8.8%; this difference was statistically significant (*p* = 0.029). There was also a significantly higher proportion of acute kidney injury in nearly one third of the patients with pacemaker implantation.

Concerning patient follow-up, it was significantly longer in the non-pacemaker-associated group (group two) (4.0 vs. 19.0 months, *p* < 0.001). In our study, there were no significant differences between the two groups regarding readmissions, TTS recurrences or all-cause death.

**Fig. 3.**
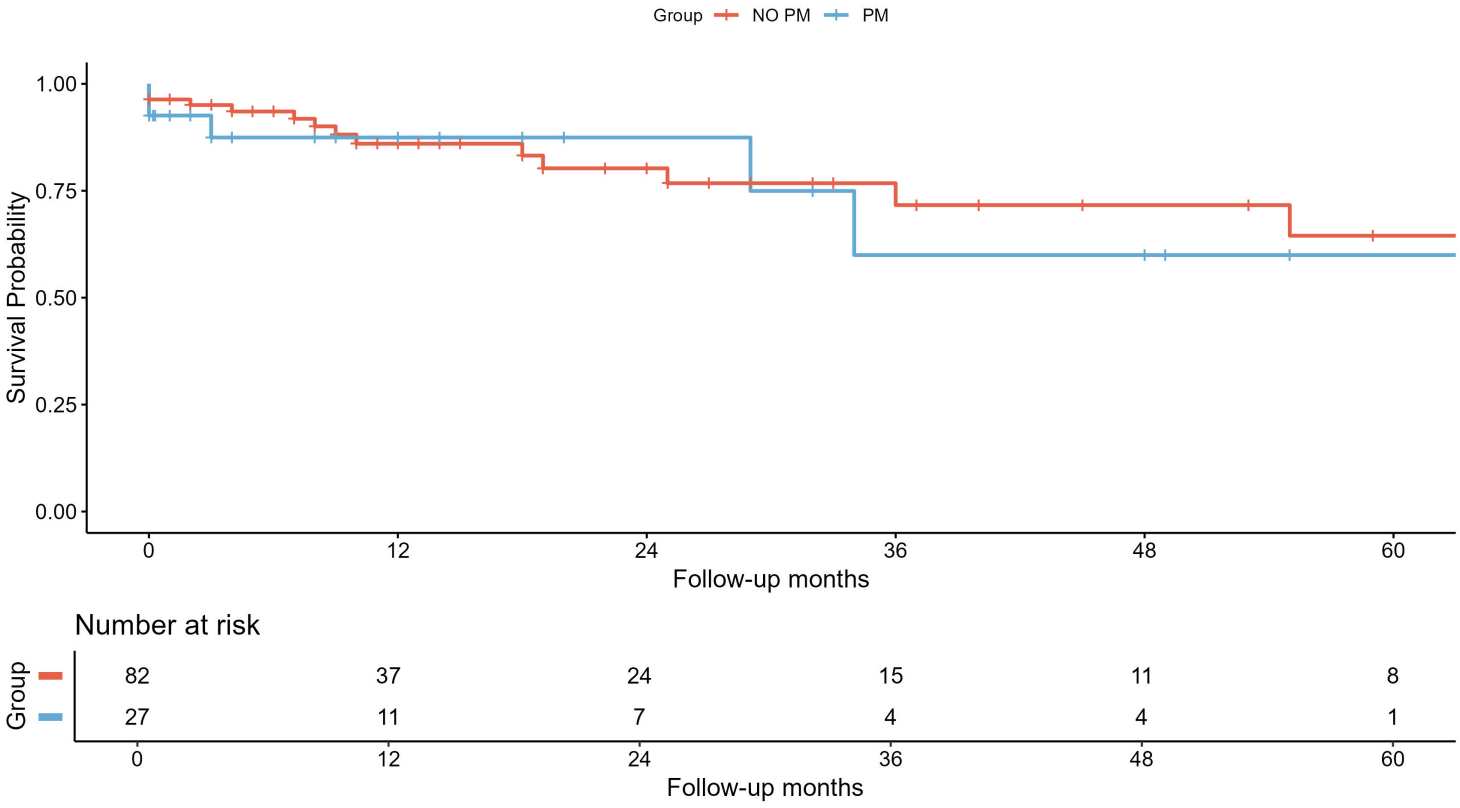
**Survival analysis of all-cause mortality using Kaplan–Meier curves**. Survival analysis of all-cause mortality after 1:3 propensity score matching represented by Kaplan–Meier curves and number of patients at risk after the end of each time period. In blue, the takotsubo syndrome (TTS) group associated with pacemaker implantation (PM) and, in red, the group not associated with pacemakers (NO PM).

Table 2 shows the comparison after 1:3 PSM, in which the differences in various cardiovascular outcomes (i.e., acute kidney injury, cardiogenic shock, LVEF and cQT interval) are lost after matching. With respect to mortality rates and TTS recurrence, no differences were observed between the two cohorts either before or after PSM. The Kaplan–Meier survival curves of all-cause mortality are presented in Fig. 3 (log-rank test: *p* = 0.670) using the PSM population.

**Table 2.** **Comparison between both groups after propensity score matching**.

		TTS due to pacemaker implantation (n = 33)	TTS not due to pacemaker implantation (n = 99)	*p*
	Age (years)	73.18 ± 8.01	70.53 ± 14.25	0.985
	Female	25/33 (75.8)	78/99 (78.8)	0.716
Comorbidities	Hypertension	22/31 (71.0)	58/99 (58.6)	0.216
	Dyslipidemia	10/33 (30.3)	23/99 (32.3)	0.829
	Diabetes Mellitus	11/33 (33.3)	24/99 (24.2)	0.306
	Smoker/Ex-smoker	8/33 (24.2)	24/99 (24.2)	1.000
	Ischemic Heart Disease^1^	2/23 (8.7)	0/92 (0.0)	0.039*
	Hepatopathy	1/21 (4.8)	16/97 (16.5)	0.302*
	Cancer	2/18 (11.1)	16/99 (16.2)	0.740*
TTS clinical presentation	Syncope	12/33 (36.4)	36/99 (36.4)	1.000
	Angina	10/33 (30.3)	24/99 (24.2)	0.491
	Vagal symptoms	10/33 (30.3)	27/99 (27.3)	0.737
	Dyspnea	15/26 (57.7)	20/67 (29.9)	0.013
	Palpitations	3/32 (9.4)	8/98 (8.2)	1.000*
	Physical/mixed trigger	33/33 (100.0)	99/99 (100.0)	-
	Apical akinesia^2^	26/33 (78.8)	79/99 (79.8)	0.901
Cardiovascular outcomes	LVEF (%)^3^	36.9 ± 7.6	41.2 ± 13.4	0.144
	cQT interval (ms)^4^	549.0 ± 76.9	521.6 ± 57.9	0.188
	Acute kidney injury	11/33 (33.3)	29/98 (20.4)	0.131
	Pulmonary edema	5/33 (15.2)	6/99 (6.1)	0.141
	Cardiogenic shock	7/26 (26.9)	16/67 (23.9)	0.760
	Intraventricular thrombus	1/33 (3.0)	5/99 (5.1)	1.000*
	Readmission	1/27 (3.7)	3/76 (3.9)	1.000*
	Follow-up (months)^5^	4.00 (1.0–29.0)	9.50 (4.0–25.5)	0.154
	TTS recurrence	0/27 (0.0)	2/77 (2.6)	1.000*
	In-hospital death	1/33 (3.0)	3/99 (3.0)	1.000*
	All-cause mortality	5/33 (15.2)	14/99 (14.1)	1.000

Categorical variables are reported as n/N (percentage). Continuous variables are presented as mean ± standard deviation or median (interquartile range), as appropriate. Pearson’s chi-square test was applied to categorical data, and the Mann-Whitney test to continuous data. Variables marked with an asterisk (*) were analysed using Fisher’s exact test. Abbreviations: TTS, takotsubo syndrome; LVEF, left ventricular ejection fraction; cQT, corrected QT interval. ^1^Regardless of its severity. ^2^Patients were classified based on whether it presented in the typical form of TTS (apical akinesia) or an atypical form (basal or mid-segment akinesia). ^3^Left ventricular ejection fraction (LVEF) is expressed as a percentage according to the biplane Simpson method by echocardiography. ^4^The corrected QT interval (cQT) is expressed in milliseconds using Bazett’s correction formula. ^5^Follow-up is expressed in months as median and interquartile range.

**Supplementary Table 2** shows the sensitivity analysis between patients with TTS following pacemaker implantation from the RETAKO registry and those obtained from the literature, which showed that patients from both subgroups were comparable in terms of their baseline characteristics and outcomes (i.e., cardiogenic shock and mortality) although a lower left LVEF was observed in the literature-derived pacemaker-associated TTS group. Therefore, we could assume that this comparison is acceptable as a whole and does not constitute a limiting bias.

With the revised PSM model (excluding angina and syncope as covariates), no statistically significant differences emerged between groups for the principal outcomes—pulmonary edema, cardiogenic shock, readmission, TTS recurrence, in-hospital mortality and all-cause mortality. However, differences persisted in secondary parameters, namely LVEF, cQT, and acute kidney injury.

## 4. Discussion

Our study included what is, to date, the largest cohort of TTS in the context of pacemaker implantation, comprising patients from a systematic review of the literature and the multicenter registry RETAKO, and compared it with a cohort of patients with TTS due to other causes, extracted from the RETAKO registry.

Our main findings were: (1) TTS in the setting of pacemaker implantation presents with a higher rate of cardiogenic shock, but not greater mortality, prior to PSM; (2) the greater rates of cardiogenic shock and acute kidney injury are lost when adjusting for physical/mixed trigger and baseline characteristics; (3) syncope is more common as an initial symptom in patients with TTS in the setting of pacemaker implantation, whereas angina is less frequent; and (4) there are no significant differences regarding sex in patients with TTS in the setting of pacemaker implantation compared to the general RETAKO cohort.

No differences were found regarding sex between TTS in the setting of pacemaker implantation compared to the general RETAKO cohort, but a trend was observed toward a higher proportion of males in the pacemaker group, which is in line with the literature [26, 27, 28]. Previous studies indicate that physical triggers, compared to psychological, are more common in male individuals, with a greater incidence of cardiogenic shock and mortality. No differences were found in mortality in our study (neither before nor after the PSM), which might be due to the limited power, owing to the uncommon nature of this particular trigger, further studies are needed to elucidate this matter.

One of the most widely accepted mechanisms in the pathophysiology of TTS is sympathetic nervous system activation with subsequent catecholamine release [29, 30]. Experimental work in animal models has demonstrated an apicaltobasal gradient in catecholamine responsiveness that reflects regional differences in adrenoceptor subtype expression [31].

Whether this sympathetic surge is triggered primarily by the pacemaker implantation itself—through procedural pain or procedurerelated anxiety—or by the advanced conduction disturbance—via low cardiac output and attendant hemodynamic stress—remains a matter of debate [17]. Reports of TTS following urgent pacemaker placement for complete atrioventricular block support the latter hypothesis [10, 14]. Conversely, occurrences of TTS following elective pacemaker placement for sicksinus syndrome in hemodynamically stable patients [15, 22], where circulating catecholamine levels are presumably low, suggest that the implantation alone may suffice as a trigger.

In fact, cases of TTS have been reported in the context of other types of percutaneous cardiac procedures, such as atrial fibrillation ablations [32] or percutaneous mitral repair with edge-to-edge therapy [33]. These interventions, associated with peri-procedural pain but not with conduction disturbances, support our hypothesis.

A further pathophysiological hypothesis is that ventricular pacing itself may be poorly tolerated and thus may trigger TTS. However, the prompt recovery of ventricular function and the low recurrence rate observed in our series—despite continued ventricular stimulation—make this explanation less likely.

Patients with non-pacemaker-associated TTS more frequently presented with angina associated with vagal symptoms, while the pacemaker-associated TTS patients were notable for a higher frequency of syncope. Núñez-Gil *et al*. [2] described primary forms of TTS presented more frequently with chest pain and vagal symptoms, but secondary TTS had a higher frequency of syncope as a clinical presentation. We believe that the initial presentation is most likely explained by the pacemaker indication (i.e., bradyarrhythmia requiring permanent pacing).

Although both groups exhibited prolonged corrected QT intervals, the duration was significantly greater in the group associated with pacemaker implantation. This finding may be attributable to the cardiac memory phenomenon as well as cQT prolongation secondary to pacing. Ventricular activation induced by pacing leads to slower depolarization and, consequently, a wider QRS complex compared to conduction through the native His-Purkinje system. These prolonged ventricular complexes are linked to abnormal repolarization, resulting in cQT interval prolongation. In several studies involving patients with cardiomyopathies, such prolongation of ventricular depolarization and repolarization has been associated with an increased risk of ventricular arrhythmias. Although TTS is not considered a true cardiomyopathy due to its reversible nature, this feature could nonetheless contribute to adverse cardiovascular outcomes in larger patient cohorts [34]. Moreover, previous studies have described an association between the risk of ventricular arrhythmias in TTS and both cQT duration [35] and Tpeak–Tend interval prolongation [36].

Rhythm disorders have been linked to decreased long-term survival in TTS [37], with cardiogenic shock being an independent predictor (either for tachyarrhythmias or bradyarrhythmias). This study aligns with our findings, as the association between a higher proportion of cardiogenic shock and TTS after pacemaker implantation (indicated mostly by bradyarrhythmias as shown in **Supplementary Table 3**) is reproduced.

Nevertheless, the short follow-up and the low rate of ventricular arrhythmias reported in the literature group preclude any conclusions to be made about the arrhythmic burden. Moreover, most of the electrocardiographic tracings are post-pacemaker implantation, and the Tpeak-Tend has not been validated in this situation. Although the electrocardiogram shows ventricular pacing with secondary repolarization alterations, this does not prevent the observation of electrocardiographic changes caused by TTS and their progression over time [38].

In the TTS group following pacemaker implantation, a significantly lower LVEF was observed, although this did not translate into a worse prognosis. We believe that, in part, the ventricular dysfunction could be explained by advanced conduction disorders, whereas another part might be attributed to contractility alterations inherent to TTS. It is likely that the ventricular dysfunction resolved quickly because the trigger was short-lived (limited to the implantation time), and the conduction disorders were resolved with pacemaker placement. This could explain the lower mortality, as it has been reported [39] that a rapid recovery of LVEF (within less than ten days) is associated with a better prognosis compared to delayed recovery. However, due to the limited follow-up in the patient cohort described in the literature, we cannot confirm this hypothesis.

In the study group, a higher percentage of cardiogenic shock was observed during the hospital stay; a phenomenon that can be observed in other particular TTS settings, such as the peripartum period [3] and the pediatric population [27], in which physical triggers are more common [26]. However, before and after adjusting for potential confounders, the mortality rate remained similar between groups. This increase in cardiogenic shock was most likely driven by the greater percentage of apical akinesia pattern and the greater LVEF dysfunction that was corrected after PSM. As in the peripartum period [3], in which the population is much younger, the detection of the TTS in a controlled in-hospital environment might have played a role in early diagnosis and prompt vasoactive therapy as well as intensive surveillance, which might have accounted for a similar mortality rate than that of the general RETAKO population, in an otherwise greater mortality setting of a physical trigger in an elderly population.

The lack of clinical data to grade cardiogenic shock according to the Society for Cardiovascular Angiography and Interventions (SCAI) scale in our series also may have influenced our inability to find differences in mortality between groups. As described by Camblor-Blasco *et al*. [40], there is an association between a higher SCAI grade in the context of TTS with higher in-hospital and one-year mortality. However, these authors report that mortality in the early stages of cardiogenic shock is low: 2.3% in SCAI A and 4.9% in SCAI B. It is possible that many of the patients in our series with cardiogenic shock had a low SCAI grade and, therefore, not a high mortality rate. Moreover, other variables that could negatively influence outcomes in cardiogenic shock in the setting of TTS, such as thyroid hormone homeostasis [41] could not be calculated due to the lack of available data.

Furthermore, in the study group, a higher rate of acute kidney injury was observed. Renal impairment in TTS has been associated with lower LVEF, higher rates of bleeding, a higher percentage of cardiogenic shock, as well as higher rates of all-cause mortality and major cardiovascular events at 5 years [42]. However, after PSM, acute kidney injury was equally common between groups, suggesting that the physical trigger was most likely driving these differences.

Although cancer prevalence was comparable in both cohorts, a longer follow-up might have revealed differences, but when truncated Kaplan-Meier curves were performed at 6, 12, 18 and 24 months (**Supplementary Fig. 1**), no differences were observed, suggesting that the shorter follow-up in the literature cases did not have a significantly impact the overall analysis.

Accurate ascertainment of malignancies in the context of TTS is clinically pertinent, as previous studies have shown that this subgroup experiences a higher incidence of adverse events [43]—both during the index hospitalization and over longitudinal follow-up.

Beta-blockers are widely used for the treatment of TTS (up to 75% in some series [2]) and have recently been shown to reduce mortality [44]. In this subgroup of patients with TTS, the use of beta-blockers appears to be a reasonably safe therapy, as the pacemaker ensures a minimum heart rate.

In the future, the potential occurrence of TTS as a complication of pacemaker implantation should be considered, particularly in relation to implant type (transvenous or leadless). However, retrospectively identifying TTS requires large databases [45].

## 5. Limitations

There is a limited amount of data available on cases of TTS associated with pacemaker implantation published in the literature. The longest series published to date, with 9 cases [6], could not be included in our work as it did not strictly meet the revised Mayo criteria because in four of the nine patients, coronary anatomy was not assessed.

Another limitation of the study is that we do not have access to a number of patients in the TTS associated with pacemaker implantation group who received isoproterenol (a drug commonly used in patients with conduction disturbances), which could act as an adrenergic trigger, thus generating potential confounding.

As this is an observational study, it is not possible to fully ensure causality. However, the 21 cases in the RETAKO registry have been thoroughly evaluated to confirm the temporal relationship between pacemaker implantation and the development of TTS. However, since we did not have access to the medical records of the 20 patients included in the systematic literature review, we were unable to reconfirm this association in this subgroup of patients.

The low recurrence rate may be explained by the limited follow-up reported in the literature; in many cases, follow-up was restricted to a single outpatient visit or ended at hospital discharge. While the recurrence rate in TTS is generally low—approximately 4% in the overall RETAKO cohort—we believe that the absence of recurrence in the pacemaker-associated TTS group is more likely due to insufficient follow-up and a small sample size than a distinctive feature of this population. Therefore, these findings should be interpreted with caution.

## 6. Conclusion

TTS following pacemaker implantation more frequently presents with syncope than with chest pain at the its initial presentation. TTS triggered by pacemaker implantation is associated with a longer cQT interval, lower LVEF, a higher rate of acute kidney injury and cardiogenic shock, with no differences in all-cause mortality or TTS recurrences, compared with TTS not associated with pacemaker implantation. However, our study shows that TTS following pacemaker implantation is associated with prognostic outcomes similar to other TTS cases secondary to physical triggers.

## Availability of Data and Materials

All the material is available through the corresponding author via e-mail upon reasonable request.

## Supplementary Material

Supplementary material associated with this article can be found, in the online version, at https://doi.org/10.31083/RCM39440.
