# Supplementary material for: Takotsubo Syndrome in the Setting of Pacemaker Implantation: Results From a Multicenter National Prospective Registry
Source: Rev Cardiovasc Med. 2025 Sep 28;26(9):39440. doi: 10.31083/RCM39440 (PMC12516771; doi:10.31083/RCM39440)
Supplement: Supplementary file 1 [file 2153-8174-26-9-39440-s1.docx]

**Supplementary material**

**Supplementary material index**

1. **Supplementary Table 1** 2

1. **Supplementary Table 2** 3
2. **Supplementary Table 3**  4
3. **Supplementary Table 4**  5
4. **Supplementary Fig. 1**  6

**Supplementary Table 1.** Clinical characteristics of patients extracted from the systematic review of the literature.

| **Article** | **Gender** | **Age** | **Indication^1^** | **Timing^2^** | **Symptoms^3^** | **ECG^4^** | **Complications^5^** | **Death** |
| --- | --- | --- | --- | --- | --- | --- | --- | --- |
| **Boghdadi 2023** | F | 73 | AVB | Few hours | Chest pain | Paced | APE | No |
| **Moinuddin 2022** | M | 65 | AVB | Few hours | Syncope | Paced/LBBB | Hypotension | No |
| **Wakatsuki 2020** | F | 81 | SSS | 1 day | Palpitations | TWI, long QT | VT, VF | No |
| **Tiritilli 2018** | F | 81 | AVB | 1 day | Chest pain, dyspnea | TWI | AHF | No |
| **Wei 2018** | F | 72 | AVB | 1 day | Dyspnea, chest pain | TWI | AHF | No |
| **Dashwood 2016** | F | 76 | SSS | 1 day | Chest pain, hypotension | TWI | Hypotension, hypoxia | No |
| **Postema 2014** | F | 61 | AVB | 1 day | Chest pain, orthopnea | Paced | AHF | No |
| **Dias 2013** | F | 72 | AVB | Few hours | Nausea, light-headedness | No info | AHF | No |
| **Kinbara 2013** | F | 69 | AVB | 3 days | Chest pain, syncope | VT | VT, VF, APE | Yes |
| **Bonfantino 2012** | F | 78 | SAB | Procedure | Chest pain, hypotension | STE | VT, SVT | No |
| **Gardini 2012** | F | 75 | AVB | Few hours | Chest pain, dyspnea | STE | No | No |
| **Mazurek 2011** | M | 77 | AVB | Few hours | Dyspnea | Paced | APE | No |
| **Brunetti 2011** | F | 65 | AVB | Few hours | Dyspnea | Paced | AHF | No |
| **Golzio (1) 2011** | F | 67 | SSS | 1 day | Chest pain | STE | No | No |
| **Golzio (2) 2011** | F | 64 | AVB | No info | Asymptomatic | STE | STE | No |
| **Kohnen 2009** | F | 83 | SSS | Few hours | Dyspnea | STE | Left ventricular thrombus | No |
| **Sham'a 2009** | F | 86 | AVB | 1 day | Acute pulmonary edema | Long QT | APE | No |
| **Chun 2007** | F | 77 | SSS | No info | Asymptomatic | TWI | No | No |
| **Kurisu (1) 2006** | F | 89 | AVB | 10 minutes | Chest discomfort | STE | AHF | No |
| **Kurisu (2) 2006** | F | 77 | AVB | 3 days | Orthopnea | STE | AHF | No |

^1^Clinical indication for pacemaker implantation.

^2^Time from pacemaker implantation to the onset of symptoms compatible with takotsubo syndrome.

^3^Symptoms associated with takotsubo syndrome.

^4^Electrocardiographic abnormalities at the time of diagnosis.

^5^Complications associated with takotsubo syndrome.

AHF: acute heart failure; APE: acute pulmonary edema; AVB: atrioventricular block; F: female; LBBB: left bundle branch block; M: male; QT: QT interval; SAB: sinoatrial block; SSS: sick sinus syndrome; STE: ST elevation; SVT: supraventricular tachycardia; TWI: T wave inversion; VF: ventricular fibrillation; VT: ventricular tachycardia.

**Supplementary Table 2.** Comparison of patients from systematic revision of the literature and from RETAKO.

|  |  | Pacemaker literature (n=20) | Pacemaker RETAKO (n=21) | p |
| --- | --- | --- | --- | --- |
|  | Age (years) | 74.4 ± 7.63 | 72.9 ± 8.11 | 0.592 |
|  | Female | 16/20 (80.0) | 16/21 (76.2) | 1.00* |
| Comorbidities | Hypertension | 11/14 (78.6) | 14/20 (70.0) | 0.704* |
|  | Dyslipidemia | 3/15 (20.0) | 8/20 (40.0) | 0.281* |
|  | Diabetes Mellitus | 5/15 (33.3) | 8/21 (38.1) | 0.769 |
|  | Smoker/Ex-smoker | 0/14 (0.0) | 8/21 (38.1) | **0.012*** |
|  | Ischemic Heart Disease^1^ | 2/6 (33.3) | 1/19 (5.3) | 0.133* |
|  | Hepatopathy | 1/2 (50.0) | 0/20 (0) | 0.091* |
|  | Cancer | 0/20 (0.0) | 2/21 (9.5) | 0.49* |
| TTS clinical presentation | Syncope | 3/20 (15.0) | 9/21 (42.9) | 0.051* |
|  | Angina | 9/20 (45.0) | 3/21 (14.3) | **0.031*** |
|  | Vagal symptoms | 6/19 (31.6) | 6/21 (28.6) | 0.836 |
|  | Dyspnea | 12/20 (60.0) | 7/14 (50.0) | 0.563 |
|  | Palpitations | 0/20 (0) | 3/20 (15.0) | 0.231* |
|  | Physical/mixed trigger | 20/20 (100) | 21/21 (100) | - |
|  | Apical akinesia^2^ | 20/20 (100) | 14/21 (66.7) | **0.009*** |
| Cardiovascular outcomes | LVEF (%)^3^ | 30.3 ± 7.7 | 40.9 ± 4.9 | **0.002** |
|  | cQT interval (ms)^4^ | 539.5 ± 0.8 | 553.2 ± 77.7 | 0.933 |
|  | Acute kidney injury | 0/20 (0.0) | 12/21 (57.1) | **<0.001*** |
|  | Cardiogenic shock | 3/20 (15.0) | 4/14 (28.6) | 0.410* |
|  | Intraventricular thrombus | 1/20 (5.0) | 1/21 (4.8) | 1.00* |
|  | Readmission | 0/19 (0.0) | 1/16 (1.5) | 0.457* |
|  | Follow-up (months)^5^ | 2.00 (1.0 – 4.0) | 16.00 (3.75 – 37.5) | **0.02** |
|  | TTS recurrence | - | - | - |
|  | All-cause mortality | 1/20 (5.0) | 4/21 (19.0) | 0.343* |

Comparison of baseline clinical characteristics, comorbidities and cardiovascular outcomes between takotsubo syndrome (TTS) associated with pacemaker implantation from systematic revision of the literature and from national multicenter registry on takotsubo syndrome (RETAKO). Abbreviations: TTS: takotsubo syndrome; LVEF: left ventricular ejection fraction; cQT: corrected QT interval

^1^Regardless of its severity.

^2^Patients were classified based on whether it presented in the typical form of TTS (apical akinesia) or an atypical form (basal or mid-segment akinesia).

^3^Left ventricular ejection fraction (LVEF) is expressed as a percentage according to the biplane Simpson method by echocardiography.

^4^The corrected QT interval (cQT) is expressed in milliseconds using Bazett's correction formula.

^5^Follow-up is expressed in months as median and interquartile range.

Categorical variables are reported as n/N (percentage). Continuous variables are presented as mean ± standard deviation or median (interquartile range), as appropriate. Pearson’s chi-square test was applied to categorical data, and the Mann-Whitney test to continuous data. Variables marked with an asterisk (*) were analysed using Fisher’s exact test.

**Supplementary Table 3.** Comparison between both groups after propensity score matching 3:1 on the following variables: dyslipidemia, diabetes mellitus, smoker/ex-smoker status, vagal symptoms, and physical or mixed trigger.

|  |  | TTS due to pacemaker implantation (n=33) | TTS not due to pacemaker implantation (n=99) | p |
| --- | --- | --- | --- | --- |
|  | Age (years) | 73.18 ± 8.01 | 72.15 ± 13.76 | 0.565 |
|  | Female | 25/33 (75.8) | 80/99 (80.8) | 0.533 |
| Comorbidities | Hypertension | 22/31 (71.0) | 68/99 (68.7) | 0.810 |
|  | Dyslipidemia | 10/33 (30.3) | 30/99 (30.3) | 1.00 |
|  | Diabetes Mellitus | 11/33 (33.3) | 33/99 (33.3) | 1.00 |
|  | Smoker/Ex-smoker | 8/33 (24.2) | 24/99 (24.2) | 1.00 |
|  | Ischemic Heart Disease^1^ | 2/23 (8.7) | 4/94 (4.3) | 0.336* |
|  | Hepatopathy | 1/21 (4.8) | 5/97 (5.2) | 1.00* |
| TTS clinical presentation | Syncope | 12/33 (36.4) | 9/97 (9.3) | **<0.001** |
|  | Angina | 10/33 (30.3) | 33/97 (34.0) | 0.695 |
|  | Vagal symptoms | 10/33 (30.3) | 30/99 (30.3) | 1.00 |
|  | Dyspnea | 15/26 (57.7) | 22/64 (34.4) | **0.042** |
|  | Palpitations | 3/32 (9.4) | 9/97 (9.4) | 1.00* |
|  | Physical/mixed trigger | 33/33 (100) | 99/99 (100) | 1.00 |
|  | Apical akinesia^2^ | 26/33 (78.8) | 75/99 (75.8) | 0.722 |
| Cardiovascular outcomes | LVEF (%)^3^ | 39.0 (35.0 – 40.0) | 45.0 (36.5 – 55.0) | **0.004** |
|  | cQT interval (ms)^4^ | 539.0 (499.5 – 598.5) | 481.0 (446.25 – 549.75) | **0.015** |
|  | Acute kidney injury | 11/33 (33.3) | 15/97 (15.5) | **0.027** |
|  | Pulmonary edema | 5/33 (15.2) | 12/98 (12.2) | 0.765 |
|  | Cardiogenic shock | 7/26 (26.9) | 12/64 (18.8) | 0.389 |
|  | Intraventricular thrombus | 1/33 (3.0) | 2/98 (2.0) | 1.00* |
|  | Readmission | 1/27 (3.7) | 9/81 (11.1) | 0.446* |
|  | Follow-up (months)^5^ | 4.00 (1.0 – 29.0) | 18.0 (7.0 – 39.5) | **0.015** |
|  | TTS recurrence | 0/27 (0) | 2/77 (2.6) | 1.00* |
|  | In-hospital death | 1/33 (3.0) | 2/99 (2.0) | 1.00* |
|  | All-cause mortality | 5/33 (15.2) | 12/99 (12.1) | 0.764 |

Categorical variables are reported as n/N (percentage). Continuous variables are presented as mean ± standard deviation or median (interquartile range), as appropriate. Pearson’s chi-square test was applied to categorical data, and the Mann-Whitney test to continuous data. Variables marked with an asterisk (*) were analysed using Fisher’s exact test. Abbreviations: TTS: takotsubo syndrome; LVEF: left ventricular ejection fraction; cQT: corrected QT interval

^1^Regardless of its severity.

^2^Patients were classified based on whether it presented in the typical form of TTS (apical akinesia) or an atypical form (basal or mid-segment akinesia).

^3^Left ventricular ejection fraction (LVEF) is expressed as a percentage according to the biplane Simpson method by echocardiography.

^4^The corrected QT interval (cQT) is expressed in milliseconds using Bazett's correction formula.

^5^Follow-up is expressed in months as median and interquartile range.

**Supplementary Table 4.** Specific indication for pacemaker implantation, pacemaker type, and implantation timing

|  | Pacemaker literature (n=20) | Pacemaker RETAKO (n=21) |
| --- | --- | --- |
| Advanced or Complete atrio-ventricular block | 14/20 (70.0) | 11/13 (84.6) |
| Sick sinus syndrome or sinoatrial block | 6/20 (30.0) | 2/13 (15.4) |
| Dual-chamber pacemaker | 18/20 (90.0) | 6/8 (75.0) |
| Single-chamber pacemaker | 2/20 (10.0) | 2/8 (25.0) |
| Emergent/Urgent implantation | 11/20 (55.0) | 11/13 (84.6) |
| Elective implantation | 9/20 (45.0) | 2/13 (15.4) |

Comparison of the specific clinical indications prompting pacemaker implantation, the type of pacemaker implanted, and procedural timing (emergent/urgent versus elective) between pacemaker-associated Takotsubo syndrome (TTS) cases identified through the systematic literature review and those documented in the national multicenter Takotsubo syndrome registry (RETAKO).


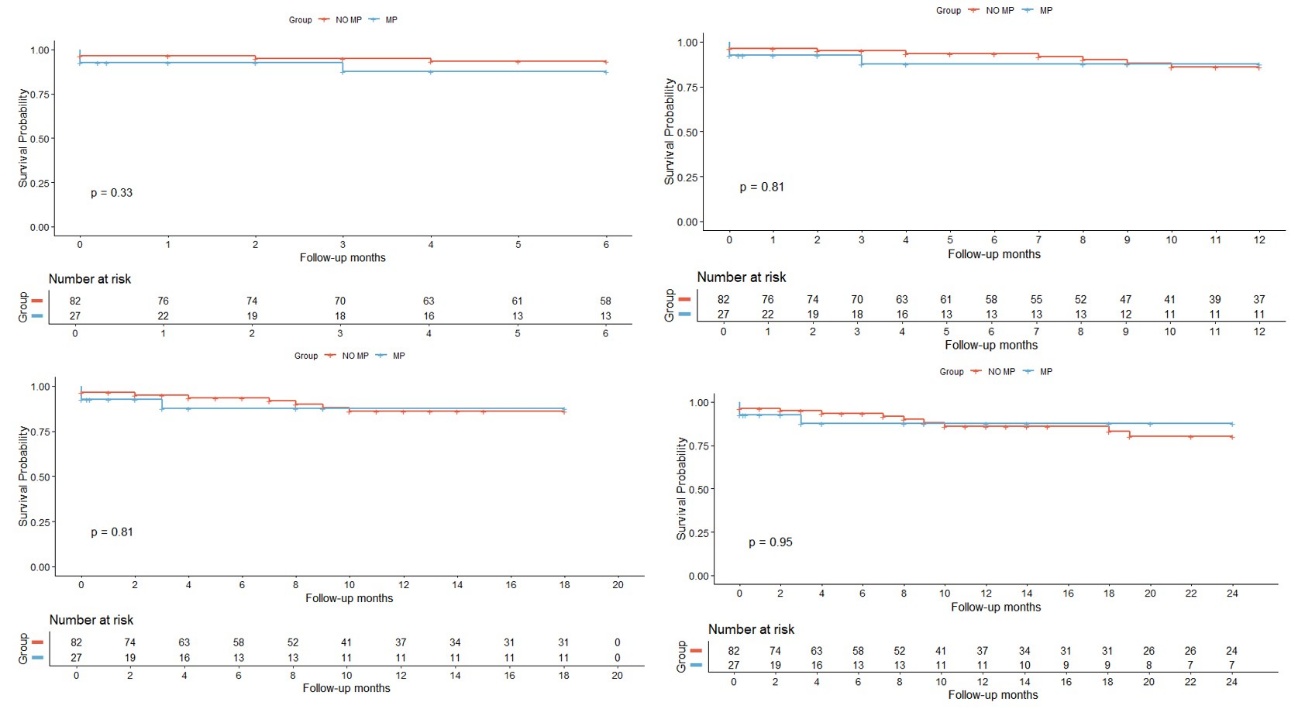
**Supplementary Fig. 1**. Truncated analysis for the pacemaker vs non-pacemaker groups to assess for potential differences in follow-up in the TTS-pacemaker group.

From upper left to downward right, we can see truncated Kaplan-Meier curves at 6, 12, 18 and 24 months which show no difference in the overall endpoint when comparing TTS-pacemaker and TTS-non-pacemaker groups.
